# Validation of diffusion tensor imaging measures of nigrostriatal neurons in macaques

**DOI:** 10.1371/journal.pone.0202201

**Published:** 2018-09-05

**Authors:** Joshua S. Shimony, Jerrel Rutlin, Morvarid Karimi, Linlin Tian, Abraham Z. Snyder, Susan K. Loftin, Scott A. Norris, Joel S. Perlmutter

**Affiliations:** 1 Mallinckrodt Institute of Radiology, Washington University School of Medicine, St. Louis, Missouri, United States of America; 2 Department of Neuroscience, Washington University School of Medicine, St. Louis, Missouri, United States of America; 3 Department of Neurology, Washington University School of Medicine, St. Louis, Missouri, United States of America; 4 Physical Therapy, Washington University School of Medicine, St. Louis, Missouri, United States of America; 5 Occupational Therapy, Washington University School of Medicine, St. Louis, Missouri, United States of America; Hudson Institute, AUSTRALIA

## Abstract

**Objective:**

Interpretation of diffusion MRI in the living brain requires validation against gold standard histological measures. We compared diffusion values of the nigrostriatal tract to PET and histological results in non-human primates (NHPs) with varying degrees of unilateral nigrostriatal injury induced by MPTP, a toxin selective for dopaminergic neurons.

**Methods:**

Sixteen NHPs had MRI and PET scans of three different presynaptic radioligands and blinded video-based motor ratings before and after unilateral carotid artery infusion of variable doses of MPTP. Diffusion measures of connections between midbrain and striatum were calculated. Then animals were euthanized to quantify striatal dopamine concentration, stereologic measures of striatal tyrosine hydroxylase (TH) immunostained fiber density and unbiased stereologic counts of TH stained nigral cells.

**Results:**

Diffusion measures correlated with MPTP dose, nigral TH-positive cell bodies and striatal TH-positive fiber density but did not correlate with *in vitro* nigrostriatal terminal field measures or *in vivo* PET measures of striatal uptake of presynaptic markers. Once nigral TH cell count loss exceeded 50% the stereologic terminal field measures reached a near zero floor effect but the diffusion measures continued to correlate with nigral cell counts.

**Conclusion:**

Diffusion measures in the nigrostriatal tract correlate with nigral dopamine neurons and striatal fiber density, but have the same relationship to terminal field measures as a previous report of striatal PET measures of presynaptic neurons. These diffusion measures have the potential to act as non-invasive index of the severity of nigrostriatal injury. Diffusion imaging of the nigrostriatal tract could potentially have diagnostic value in humans with Parkinson disease or related disorders.

## Introduction

Diffusion tensor imaging (DTI) and diffusion tensor tractography (DTT) have been established as sensitive and valuable methods for probing microscopic changes in brain tissue in health and disease states. Numerous applications for these methods have been demonstrated in both clinical and research settings [1]. However, the interpretation of these methods remains uncertain, since the microscopic changes that underlie the observed macroscopic DTI changes often remain unclear [2]. In particular, measuring quantitative change in DTI measures is challenging in areas of brain with many crossing fibers.

The potential challenge posed by crossing or diverging/converging fibers may limit application of these methods for quantification of the nigrostriatal pathway. This dopaminergic (DA) pathway degenerates in people with Parkinson disease (PD) which causes the initial motor manifestations of the disease. Objective measures of the integrity of this pathway could be useful for testing new therapies that may slow PD progression. Molecular imaging methods have focused on measurements of striatal uptake of various radioligands targeting presynaptic function such as [^18^F]-6-fluorodopa (FD) (which primarily reflects presynaptic decarboxylase activity and subsequent storage), [^11^C]dihydrotetrabenazine (DTBZ) (which has specificity for vesicular monamine transporter type 2 [VMAT2]) and a variety of membranous presynaptic dopamine transporter (DAT) radioligands including [^11^C]carbomethoxy-3beta-(4-flurophenyl)tropane (CFT) [3, 4]. Striatal uptake of these various radioligands reflects severity of nigrostriatal injury but only when less than 50% of nigral DA neuronal cells bodies are lost; At that point, these terminal field biomarkers have reached a nadir and no longer reflect increasing nigrostriatal injury, whereas nigral cell body injury fully reflects the subsequent severity of parkinsonism[3–5]. Thus, a measure of nigrostriatal fiber integrity could provide an alternative measure of this dopaminergic system.

In the current study we used a non-human primate (NHP) animal model with varying degrees of nigrostriatal injury produced by a single internal carotid infusion of graded doses of 1-methyl-4-phenyl-1,2,3,6-tetrahydropyridine (MPTP), toxin selective for dopaminergic neurons. This model permits cross modality comparisons of dopaminergic biomarkers using *in vivo* MRI DTI measures with *in vivo* PET measures, and behavioral ratings of motor parkinsonism. *In vitro* measures include counts of nigral cell bodies of dopaminergic neurons in the substantia nigra and striatal DA fiber length density, which reflects axonal integrity [6]. Additionally, terminal field measures of these neurons in the striatum include striatal dopamine concentration, immunohistochemical measures of dopamine transporter (DAT) varicosities, and terminal arbor densities [5, 6]. Table 1 summarizes the multiple measures investigated in this study and their association with either the nigrostriatal track or the terminal fields of the striatum. Our hypothesis in the current study was that DTI and DTT measures would correlate with severity of nigrostriatal injury defined by loss of *in vitro* measures of nigral dopaminergic (DA) neurons or striatal fiber length density but not provide comparable information with either *in vivo* PET or *in vitro* histological terminal field measures in striatum.

**Table 1:** Summary of nigrostriatal measures.

	Substantia Nigra and Nigrostriatal tract	Terminal Fields in the Striatum
*Ex vivo*/Stereology	Nigral cell count Striatal fiber length density	Striatal Dopamine Striatal DAT Varicosity Density Striatal Terminal Arbor Density
Radioligands/PET	--	[^18^F]-6-fluorodopa (FD) [^11^C]dihydrotetrabenazine (DTBZ) [^11^C]carbomethoxy-3beta-(4-flurophenyl)tropane (CFT)
Diffusion MRI	Substantia Nigra MD, FA Nigrostriatal track MD,FA	Putamen MD, FA

## Materials and methods

### Monkey husbandry

Sixteen male macaque nemestrina (mean age = 5.4 +/- 1.0) were housed in the nonhuman primate facility of Washington University of St. Louis (WU), and were studied according to requirements of the NIH, and after approval of the Institutional Animal Care and Use committee (IACUC) at WU. The protocol numbers have been 20170100, 20140128, 20110161 (renewed every three years and the last approval date was July 6, 2017). All animals were obtained from licensed nonhuman primates facilities with the assistance and oversight of the Division of Comparative Medicine at Washington University. All procedures were designed to conform to suggestions of the 2006 Weatherall Report “The use of non-human primates in research” with steps taken to ameliorate subject suffering in accordance with the requirements of the NIH. Animals were maintained in facilities with 12 hour dark light cycles, were given access to food and water ad libitum, and provided with a variety of psychologically enriching tasks such as watching movies or playing with appropriate toys to avoid effects of sensory deprivation. The NHPs were monitored daily by the researchers, animal care staff and a veterinarian to check their general health and welfare. Each animal had baseline training and scans. All procedures including scans and administration of MPTP were done under anesthesia. Humane endpoints were pre-defined in this protocol; however, death was not an endpoint of this study, and none of the animals in this report were euthanized prior to the planned time point. After MPTP lesioning, we continued behavioral observation and imaging data collection for 2 months after which we performed euthanasia to permit analysis of in vitro measures to compare with *in vivo* measures. After the last scan, each animal was euthanized with pentobarbital at least 150 mg/kg IV, the brain was rapidly removed and tissue was prepared, stained, and counted [7].

### Lesion methods

Monkeys were scanned with MRI and PET prior to lesioning. After overnight fasting with access to water up to two hours before the procedure, animals were initially sedated with ketamine 10–20 mg/kg i.m. and given gluycopyrrolate to reduce secretions. At that point, animals were intubated with a soft cuff endotracheal tube and then anesthesia was maintained with Isoflurane inhalation (1–3%) with oxygen; thus there was no need for restraints. MPTP (Sigma, St. Louis, MO; 0.1 mg/ml in normal saline) with doses varying from 0 to 0.31 mg/kg was infused no faster than 1 ml per minute into the right internal carotid artery under angiographic control when the animals were anesthetized, as described elsewhere [7, 8]. MPTP dose was varied to induce different degrees of neuronal injury. Angiograms before and after the infusion documented maintenance of proper catheter position and good distal blood flow. Proper safety procedures were followed for handling MPTP and all contaminated tissues and waste products [8]. MPTP was given to only one side of the brain to produce contralateral parkinsonism which permitted all animals to care for themselves (defined as able to eat and drink independently, move around the housing facility without difficulty without apparent discomfort) and received no dopaminergic drugs at any time. PET and MRI scans were repeated at a minimum of 4 weeks after treatment with MPTP to allow for resolution of acute effects [6].

### Behavioral evaluations of parkinsonism

Each animal was videotaped several times during the period 3–5 months before and up to 2 months after MPTP treatment for behavioral ratings. The videos were scored by the same observer who was blinded to the MPTP dose. The ratings focused on parkinsonian features (bradykinesia, tremor, and flexed posturing) using a previously validated scale (0 to 18 per side) that was developed specifically for non-human primate studies [8, 9]. Further details of behavioral ratings are provided in prior publications [6].

### PET scans

Three PET radiotracers were used to assess presynaptic dopaminergic nigrostriatal neurons: FD, DTBZ and CFT. Animals were anesthetized [8] and scanned on a Siemens Microsystems (Knoxville, TN) MicroPET Focus 220 scanner [10]. A transmission scan was followed by IV injection of 7.7–10.4mCi of DTBZ, 7.7–10.4mCi of CFT, or 3-10mCi of FD. PET data were collected for 120 minutes for all tracers. There was a 3 hour gap between each radiopharmaceutical injection, with FD being the last in accordance with the longer half-life of [^18^F] (110 min versus 20 min for [^11^C]). No correction of intra-scan motion was needed since the anesthetized animal’s head was immobilized over the duration of each scan following radiopharmaceutical injection. PET data were acquired over multiple days, as previously described [3]. The first acquired PET scan defined a baseline. To assess interval change, PET images acquired on subsequent days were co-registered to the baseline.

### PET analysis

All volume of interest (VOI) analysis was done by investigators blinded to the clinical status of the monkeys. Caudate, putamen and a reference occipital region were manually traced as VOIs on structural T1w MRI images of each monkey. For each monkey, the structural image was registered to the target PET scan using a previously described technique [11]; hence, all structural and PET emission data were mutually in register. Tissue time-activity curves were evaluated in the traced VOIs [11]. To reduce volume averaging with neighboring regions the VOIs were eroded to include only voxels with at least 90% of the maximal counts within that VOI. After extracting the tissue activity curves, we calculated the influx constant (K_OCC_) for FD data [12], and the non-displaceable binding potential (BP_ND_) for CFT and DTBZ [13]. These PET data have been reported previously[3].

### MRI methods

The MRI obtained prior to lesioning was not used in this study. MRI scanning on each NHP was performed under anesthesia at least 4 weeks after MPTP (mean = 59 days, range 30–123 days) to allow for resolution of any acute effects after MPTP treatment. All scans were done on a Siemens Trio 3T scanner with a knee coil. Anatomical scans were obtained using T1-weighted (T1w) MPRAGE sequence (voxel size 0.8 mm^3^; TR/TE/TI 1900/3.5/1000 ms; IPAT 2) and T2-weighted (T2w) SPACE sequence (voxel size 0.8 mm^3^; TR/TE 3200/243 ms; IPAT 2). Diffusion imaging was performed using a spin-echo EPI sequence (voxel size 1.1 mm^3^; TR/TE 6200/74; IPAT 2) with 60 directional vectors with a b value of 1000 s/mm^2^ and 6 baseline images with b value of 0. The diffusion scan was repeated 8 times to improve signal to noise.

All individual diffusion images were internally registered to the b0 image, which in turn was registered to the T2w image with stretch and shear enabled (12-parameter affine transform) to partially compensate for echo planar imaging (EPI) distortion. The T2w images then were registered to the individual T1w images using a multimodal registration with vector gradient metric maximization [14]. Finally, the T1w images were registered to an atlas representative macaque nemestrina target produced by mutual co-registration of the MPRAGE images from 10 of the monkeys prior to lesioning. Algebraic composition of transforms enabled resampling of any data type in register with any other. Thus, regions of interest (ROI) generated on anatomical images could be applied in register with the DTI data to permit DTI parameter measurement and tracking. These techniques were implemented as described in humans [14]. The diffusion tensor in each voxel was computed using the FSL diffusion toolbox [15] and derived measures of mean diffusivity (MD), axial diffusivity (AD), radial diffusivity (RD), and fractional anisotropy (FA) were computed in each voxel of the brain.

### DTI tracking

**Fig 1:**
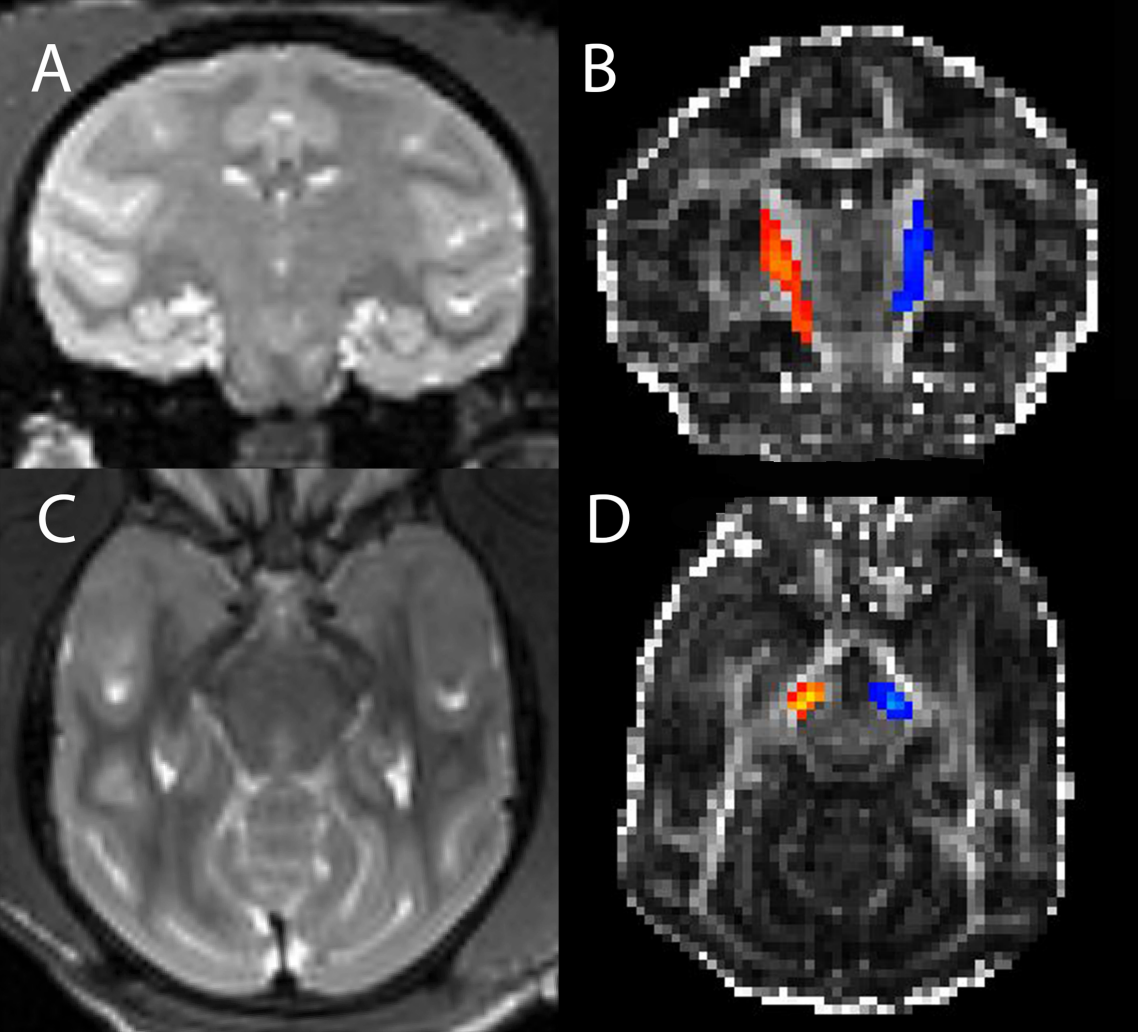
Nigrostriatal track volume. T2 weighted coronal image through the striatum (A) and FA weighted image (B) with superposed nigrostriatal track volumes (right in orange and left in blue) in a single monkey. Analogous transverse images at a level above the substantia nigra are shown in (C) and (D).

DTI probabilistic tracking was performed using the FSL package (Oxford, UK) [15, 16] to identify the nigrostriatal tracts. An expert macaque anatomist (MK) placed ROIs in the bilateral substantia nigra (SN) and the putamen (PUT) of the macaque atlas. These ROIs were transformed back into each monkey’s individual DTI space for the purpose of DTI parameter sampling and tracking. Each ROI position was inspected for accuracy by an experienced operator and a neuroradiologist (JR, JSS). ROI positioning was minimally adjusted for each individual macaque based on visual inspection. DTI tracking was performed separately on the left and on the right side of each monkey brain between the SN and PUT. Sample track volumes in a single monkey are demonstrated in Fig 1. Following tracking between the two regions an optimal tract volume was created by selecting a threshold of 0.03. In the final step of the DTI processing, DTI parameter values were sampled within the track volume and within each of the ROIs.

### *Ex vivo* methods

Striatal dopamine was measured using high-performance liquid chromatography (HPLC) with electrochemical detection [7, 17] and expressed as nanograms per gram of brain tissue. Unbiased stereological counts of nigrostriatal neurons were done on tyrosine hydroxylase (TH) immunostained midbrain slices [7]. Only TH positive neurons were counted. There were no TH negative nigral cells as judged by morphology with cresyl violet counter staining. Striatal fiber length density, immunohistochemical measures of dopamine transporter (DAT) varicosities and terminal arbor densities were measured with stereologic methods, as previously described[5]. These *in vitro* data have been reported previously[5, 6].

### Statistical methods

For all measurements, we calculated a ratio of MPTP-injected over control side (right over left) to account for global inter-subject variation. Results were analyzed with Matlab (Natick, MA). Linear regression was performed between the DTI parameters and the PET and histological data obtained in each macaque.

## Results

All the results plotted below are ratios of the value in the right lesioned side divided by the control left side. The only exceptions to this are the parkinsonism motor ratings and the MPTP dose.

### Results of the MD in the nigrostriatal tract

**Fig 2:**
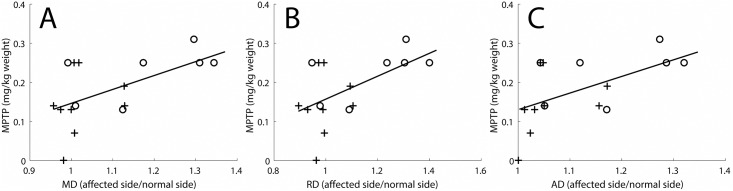
Diffusivity measures correlated with MPTP dose. Diffusivity ratios (dimensionless, affected side divided by normal side) correlated with MPTP dose (mg/kg body weight). The diffusivity measures include (A) Mean diffusivity (R^2^ = 0.33, p = 0.02), (B) Radial diffusivity (R^2^ = 0.31, p = 0.02), and (C) Axial diffusivity (R^2^ = 0.32, p = 0.02). Crosses indicate monkeys in which there was less than 50% loss of nigral cell bodies, and circles indicate monkeys in which there was a greater than 50% loss of nigral cell bodies.

MPTP dose correlated with diffusivity parameters in the nigrostriatal tract (Fig 2). Monkeys with nigral cell body loss of less than 50% are represented with crosses, and monkeys with greater than 50% cell loss with circles. This convention is kept in the subsequent figures. There was no correlation of MPTP dose with FA values. Note that the larger MPTP doses correspond to increased mean diffusivity (MD) values on the affected side as compared to the control side. In the following analyses, diffusivity measures constitute the primary DTI results.

**Fig 3:**
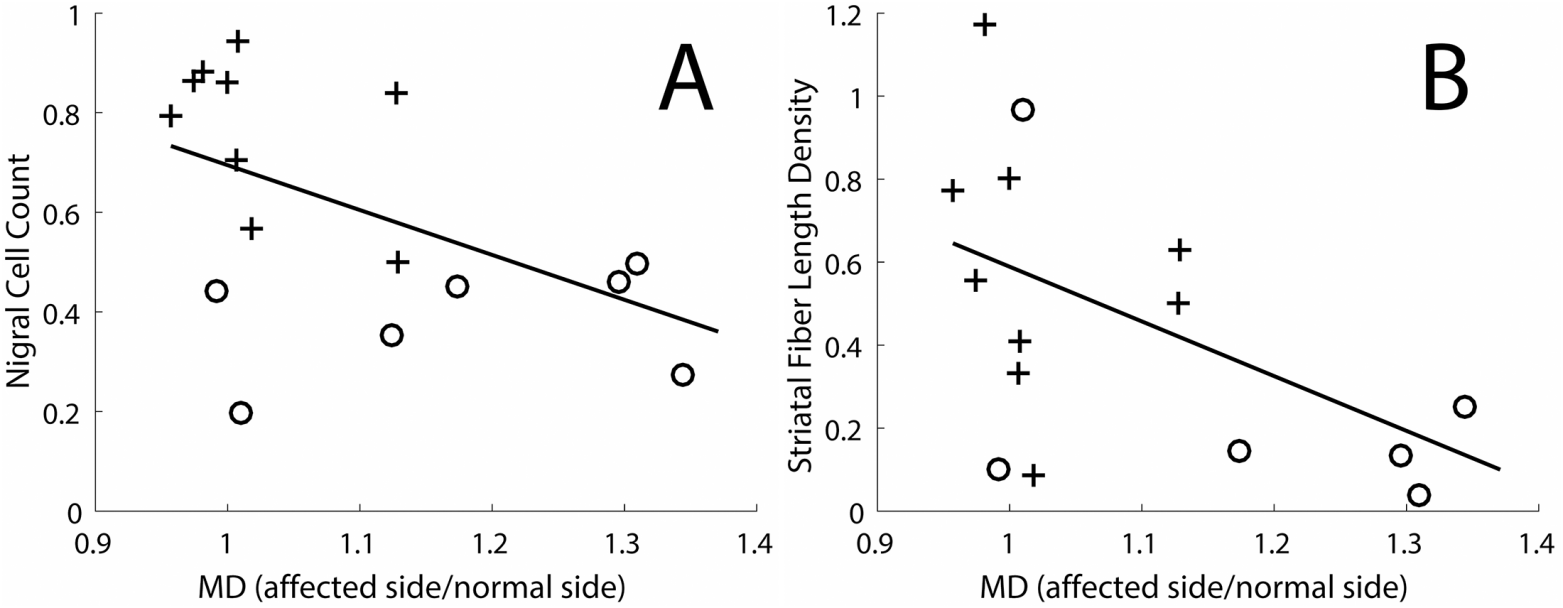
Nigral cell count and striatal fiber length density as a function of MD in the nigrostriatal tract. (A) Nigral cell count as a function of the MD in the nigrostriatal tract (R^2^ = 0.28, p = 0.03). (B) Striatal fiber length density as a function of the MD in the nigrostriatal tract (R^2^ = 0.29, p = 0.04). Crosses indicate monkeys in which there was less than 50% loss of nigral cell bodies, and circles indicate monkeys in which there was a greater than 50% loss of nigral cell bodies.

The *in vivo* MD measures in the nigrostriatal tract correlated with the *in vitro* unbiased stereologic counts of TH-stained nigral cell count and the striatal fiber density (measured by stereology of TH-stained fibers) (Fig 3). The increase in MD in the nigrostriatal tract continued to increase throughout the range of either nigral cell loss (reflecting cell bodies in the substantia nigra, Fig 3A) or striatal fiber density (reflecting nigrostriatal tract fibers throughout the striatum, Fig 3B).

**Fig 4:**
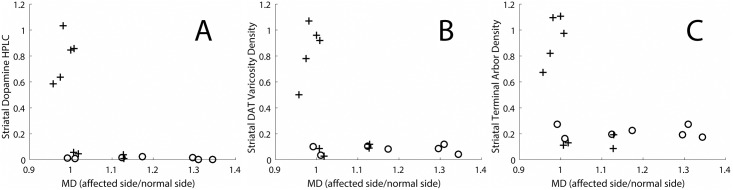
Histological measures of terminal fields of nigrostriatal neurons in the striatum as a function of the MD in the nigrostriatal tract. The measures include (A) HPLC quantification of striatal dopamine measures, (B) Stereological measure of DAT varicosity density, (C) Average terminal arbor density. Crosses indicate monkeys in which there was less than 50% loss of nigral cell bodies, and circles indicate monkeys in which there was a greater than 50% loss of nigral cell bodies.

In contrast, *in vivo* MD measures in the nigrostriatal tract did not clearly correlate with *in vitro* histological measures of terminal fields of nigrostriatal neurons in the striatum. Once the loss of nigral cells bodies exceeded 50% (cross signs in Fig 4), the *in vitro* histological terminal field measures reached a floor while the MD measures continued to increase. These *in vitro* measures include HPLC-based quantification of striatal dopamine concentration (Fig 4A), stereological-based measures of DAT varicosity density (Fig 4B) and the average terminal arbor density measures (Fig 4C). These plots demonstrate that, when the MD values on the affected side exceed those on the control side by greater than 10% (MD>1.1 on the x-axis of the plots), the striatal dopamine measures reach a maximal reduction (floor effect).

**Fig 5:**
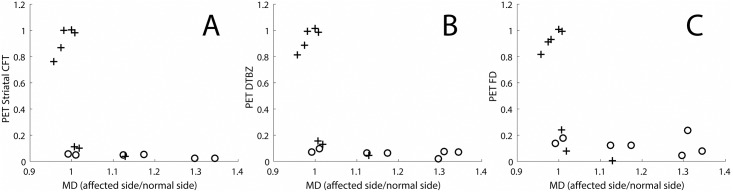
In-vivo PET measures of pre-synaptic markers as a function of the MD in the nigrostriatal tract. The measures include (A) Striatal CFT (a DAT marker), (B) DTBZ (a VMAT2 marker), (C) FD (a decarboxylase marker). Crosses indicate monkey’s in which there was less than 50% loss of nigral cell bodies, and circles indicate monkeys in which there was a greater than 50% loss of nigral cell bodies.

*In vivo* MD measures in the nigrostriatal tract have the same relationship to *in vivo* PET measures of presynaptic markers as they do to *in vitro* histologic measures of terminal fields (Fig 5). In this case, the MD measures increased as the *in vivo* terminal field measures initially declined. However, once the striatal PET measures reached near zero (again corresponding to about a 50% loss of nigral cell bodies), the MD measures continued to increase despite no further change in the striatal PET measures. Striatal CFT (a DAT marker, Fig 5A), DTBZ (a VMAT2 marker, Fig 5B) and FD (primary a decarboxylase marker, Fig 5C) changed in the same fashion as shown in Fig 4.

### Results of other DTI parameters in the nigrostriatal tract

The other diffusivity parameters in the nigrostriatal tract (AD and RD) demonstrated findings similar to the MD. Thus, Fig 2B and 2C demonstrate statistically significant linear correlations between the AD and RD and MPTP dose (p = 0.02 in both cases). Plots of AD and RD analogous to Fig 3 also demonstrated statistically significant linear correlation between the AD and RD and nigral cell counts as well as striatum fiber length density (p< = 0.04 in all cases) (S1 Fig). The floor effect was well visualized with these variables in plots analogous to Figs 4 and 5 (S2 and S3 Figs). There was no consistent relation between the FA measure and the diffusivity measures and no FA floor effect.

### Results of DTI parameters in the regions of the SN and striatum

In addition to the above reported analysis in the nigrostriatal tracts, we repeated this analysis for the end points of the tracts, selecting ROIs in the SN and the striatum. No statistically significant effects were noted in any of the DTI measurements in the SN and striatum ROIs. A weaker floor effect was noted in these areas in the diffusivity measures (MD, RD, and AD).

Measures of MD in the nigrostriatal tract did not correlate with motor ratings of parkinsonism. The ratings used were those that most closely corresponded in time with the MRI-based DTI measures and not those previously published [6].

## Discussion

We report that MD measures in the nigrostriatal tract of non-human primates exposed to MPTP correlate with the degree of *in vitro* measures of nigral cell body injury or distal striatal fiber length density. Numerous prior studies have demonstrated the underlying microscopic basis for the diffusion tensor signal and the ability to infer microstructural information from DTI data [1]. In our data, MD measures of the nigrostriatal tract correlated throughout the range of deficits with nigral cell bodies and striatal fiber length density (Fig 3). Thus, MD measured in the nigrostriatal tract likely reflects the integrity of nigrostriatal cell bodies and axons (measures located in the left column, Table 1). These results provide direct validation of *in vivo* MRI-based MD diffusivity measures of the nigrostriatal tract.

The *in vivo* MRI-based MD measures do not correlate with the striatal terminal field measures (measures located in the right column, Table 1), whether obtained *in vivo* with PET radioligands targeting presynpatic nigrostriatal neurons (Fig 5) or *in vitro* measures of striatal terminal fields (Fig 4). With greater nigral cell loss, the DTI based MD measures of the nigrostriatal tract continue to increase despite the lack of change in the near zero striatal values of the *in vitro* and *in vivo* striatal terminal field measures. This rather marked distinction supports the notion that the nigral cell bodies and striatal fiber length density measures change together with injury of the nigrostriatal tract whereas the terminal field measures of the nigrostriatal tracts reach a floor with respect to cell body and fiber density measures. We reported this same floor effect comparing *in vitro* histological data with striatal dopamine and the nigral cell counts as well as between *in vivo* PET measures of terminal fields with CFT, DTBZ or FD compared to nigral cell counts [3]. Our data provide no evidence that MD measures in the nigrostriatal tract reflect terminal field measures, whether measured by *in vitro* or *in vivo* methods.

There was no evidence of a floor effect with respect to motor parkinsonism and measures of nigral cell count or striatal fiber length density. One might anticipate that the *in vivo* MD measures would correlate with the *in vitro* striatal fiber length density measures since both reflect the fibers connecting nigral cell bodies and striatal terminal fields. Indeed, we did find a moderate correlation between the MD measures and the striatal fiber length density (Fig 3B). The *in vivo* DTI measures were derived from neuroimaging measures between the substantia nigra and striatum, whereas, the *in vitro* striatal fiber density measures included data only from TH fibers in the striatum. The degree of loss in these two locations and the potential differences in sensitivity of these two measures could contribute to some variability. In fact, degeneration of the nigrostriatal tract may occur through axonal degeneration in PD [18, 19] and may be even more likely in MPTP-induced injury since MPTP gains entry to dopaminergic cells through DAT [20]. A similar analysis applies to comparison of the MD measures in the nigrostriatal tract with *in vitro* stereologic measures of TH stained cell bodies in the substantia nigra (Fig 3A). The relatively stronger correlation between MPTP dose and the *in vivo* MD measures in the nigrostriatal tract (Fig 2) probably reflects the general degree of injury produced by MPTP that may occur throughout the nigrostriatal pathway. The lack of correlation between the DTI measures and severity of parkinsonism may reflect time-dependent degeneration not accounted for in the acquired MRI data. We previously reported that behavioral measures correlate well with nigral cell counts of TH-stained neurons when collected at nearly the same time, two months after MPTP [6]. At this time, all behavioral measures were quite stable; our preliminary data suggest that this is when the *in vitro* measures also are stable. In contrast, in the current study, these *in vitro* and behavioral measures were collected at different times.

*Ex-vivo* histological correlation with DTI values has been demonstrated in various models of neuronal fiber tissue, including demyelinating and dysmyelinating disease [21], models of traumatic brain injury in mice [22], spinal cord lesions in humans [23], and in multiple other animal models, although, to our knowledge, such comparisons have not been done for the nigrostriatal tract, an area with potentially confounding crossing fibers, and an area that is particularly pertinent to progression of PD.

Our study has relevance to investigations in people with PD. Degeneration of the nigrostriatal tract contributes to motor manifestations in animal models of PD [6] and to people with PD [19, 24]. Thus, DTI potentially provides an *in vivo* MRI-based method for identifying early degeneration that may occur in PD. The sensitivity and specificity of this approach remain to be determined. A study by Ofori et al. [25] used a novel bi-tensor diffusion model to evaluate the free water content in the substantia nigra in a group of 25 patients with PD and a control group. These subjects were followed over a 1 year period. Free water values were found to be stable in the control group but increase in the PD group and also predict changes in bradykinesia scores and cognitive status. These findings were further confirmed in a larger multi-site cohort that was followed over a 4 year period [26]. These results are consistent with our study since both the MD (average diffusion in the voxel) and free water (a measure of the extracellular isotropic volume fraction) are related measures, although free water content is a more sensitive measure. Meijer et al. [27] recently reviewed prior cross-sectional studies of PD with DTI used various forms. Some of the reviewed studies reported decreases in FA [28–30] (not seen in our study), and all but one reported increased diffusivity in PD [29] (similar to our findings), although direct comparison with our results is difficult due to differences in methodology. All of these reviewed studies compared patients with PD (and often some atypical variants) vs. a healthy control group, which experimental design differs from that used in our MPTP study. A more recent study by Tan et al. [31] compared DTI values in the nigrostriatal tract in 21 patients with PD with those in 19 healthy controls using deterministic tractography. This methodology is similar but not identical to our use of probabilistic tractography. They demonstrated significant differences in DTI parameter values in the nigrostriatal tract including lower FA (not seen in our study) and higher diffusivity values in the MD and RD, but not AD. Our study demonstrated higher diffusivity in all diffusivity measures. The discrepancy in the FA values between our study (no change) and the decrease in FA reported in Tan et al. and other studies may relate to the difference between human PD and the focal, MPTP induced PD symptoms measured in our study. Another possible reason for these discrepancies in the PD literature is that changes in the free water extracellular compartment in SN found using a bi-tensor model are confounded using a standard DTI analysis[32]. It is possible that our positive results reflect use of probabilistic tractography which involves a more complicated algorithm than the standard single tensor model [16].

DTI of the nigrostriatal tract potentially provides an objective measure of severity of nigrostriatal injury. This technique could help to assess efficacy of new therapies, particularly in early PD. Routine clinical application may or may not be feasible. The relationship of the MD measures in the nigrostriatal tract with nigral cell counts suggests that this measure also may reflect the full range of nigrostriatal injury, a major advantage compared to molecular imaging methods focused on presynaptic measures in the striatum [3]. Although some MR-based measures, like the bi-tensor model, are not changed by drugs used for symptomatic treatment [33] this remains to be proven for our method. Longitudinal measures in people with PD will be needed to determine whether our methods reflect disease progression, as others have done for bi-tensor measures in SN [26]. Further studies will be required to fully evaluate this method in human populations.

## Supporting information

S1 Fig Nigral cell count and striatal fiber length density as a function of RD and AD in the nigrostriatal tract. (A,C) Nigral cell count as a function of the RD and AD in the nigrostriatal tract. (B,D) Striatal fiber length density as a function of the RD and AD in the nigrostriatal tract (R^2^ = 0.29, p = 0.04). Crosses indicate monkeys in which there was less than 50% loss of nigral cell bodies, and circles indicate monkeys in which there was a greater than 50% loss of nigral cell bodies.
(TIF)

S2 Fig Histological measures of terminal fields of nigrostriatal neurons in the striatum as a function of the RD and AD in the nigrostriatal tract. The measures include (A,D) HPLC quantification of striatal dopamine measures, (B,E) Stereological measure of DAT varicosity density, (C,F) Average terminal arbor density. Crosses indicate monkeys in which there was less than 50% loss of nigral cell bodies, and circles indicate monkeys in which there was a greater than 50% loss of nigral cell bodies.
(TIF)

S3 Fig In-vivo PET measures of pre-synaptic markers as a function of the RD and AD in the nigrostriatal tract. The measures include (A,D) Striatal CFT (a DAT marker), (B,E) DTBZ (a VMAT2 marker), (C,F) FD (a decarboxylase marker). Crosses indicate monkey’s in which there was less than 50% loss of nigral cell bodies, and circles indicate monkeys in which there was a greater than 50% loss of nigral cell bodies.
(TIF)
