# Supplementary figures and images for: Validation of diffusion tensor imaging measures of nigrostriatal neurons in macaques
Source: PLoS One. 2018 Sep 5;13(9):e0202201. doi: 10.1371/journal.pone.0202201 (PMC6124722; doi:10.1371/journal.pone.0202201)

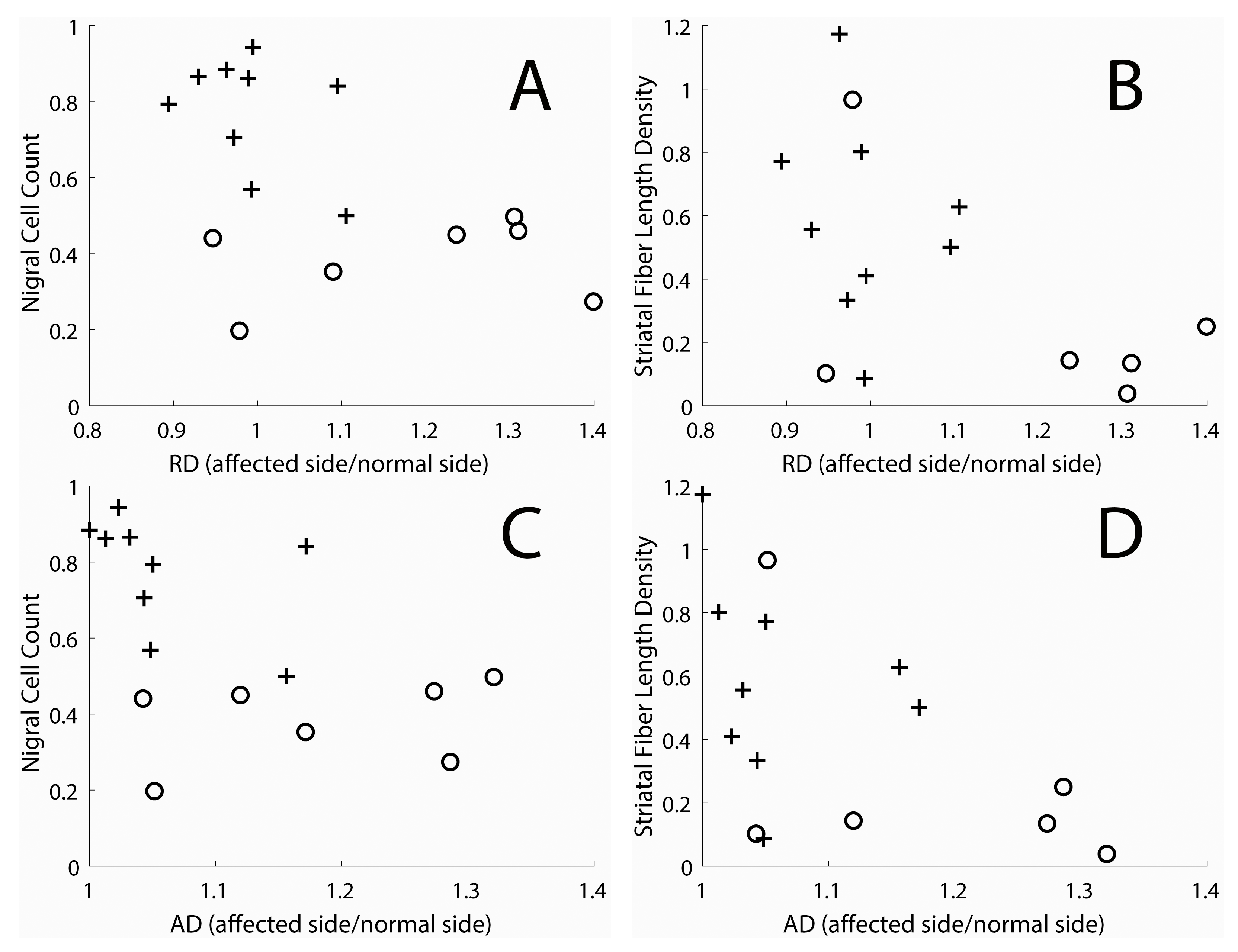

Supplement: S1 Fig — (A,C) Nigral cell count as a function of the RD and AD in the nigrostriatal tract. (B,D) Striatal fiber length density as a function of the RD and AD in the nigrostriatal tract (R2 = 0.29, p = 0.04). Crosses indicate monkeys in which there was less than 50% loss of nigral cell bodies, and circles indicate monkeys in which there was a greater than 50% loss of nigral cell bodies. (TIF) [file pone.0202201.s001.tif]

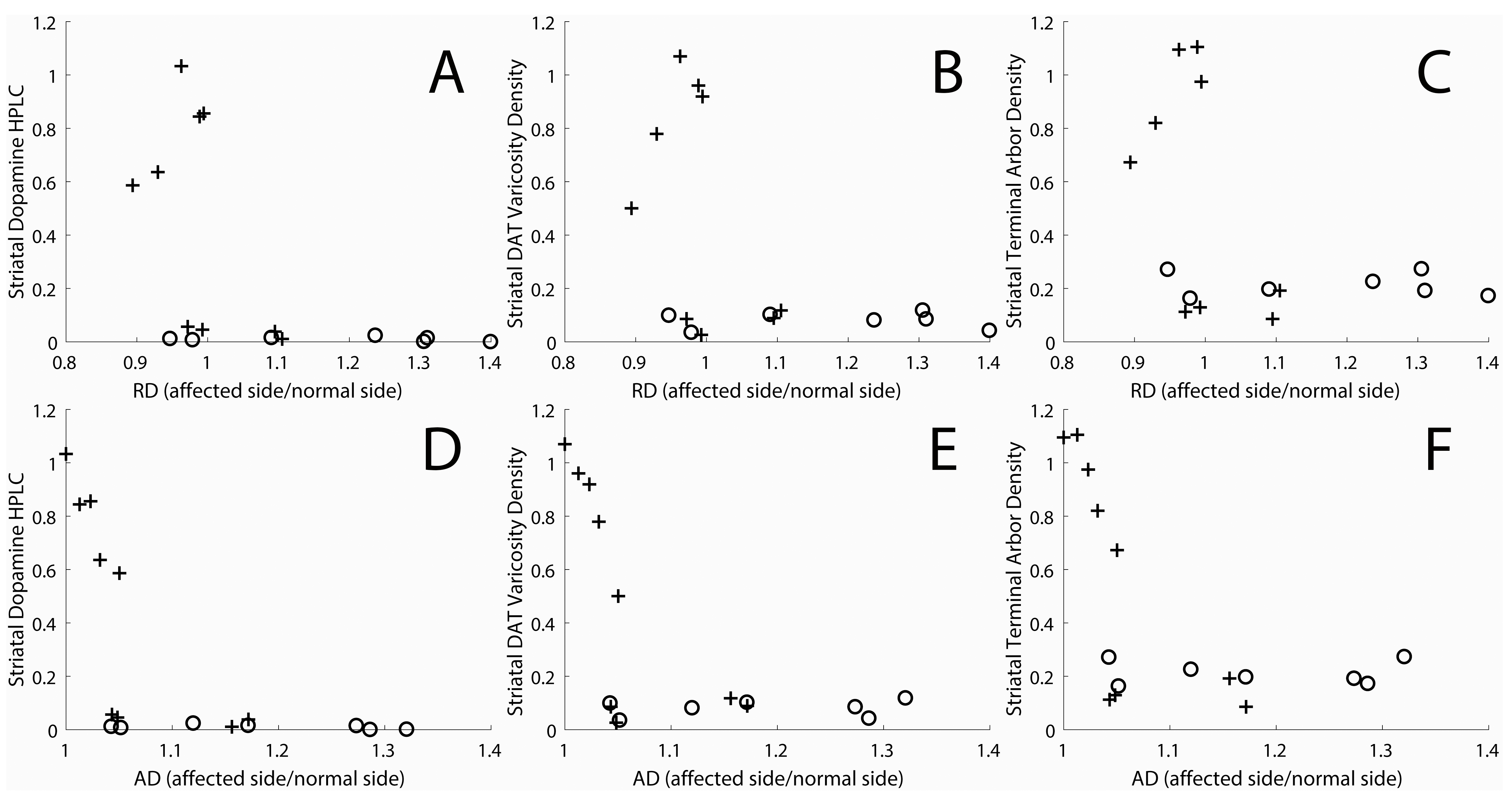

Supplement: S2 Fig — The measures include (A,D) HPLC quantification of striatal dopamine measures, (B,E) Stereological measure of DAT varicosity density, (C,F) Average terminal arbor density. Crosses indicate monkeys in which there was less than 50% loss of nigral cell bodies, and circles indicate monkeys in which there was a greater than 50% loss of nigral cell bodies. (TIF) [file pone.0202201.s002.tif]

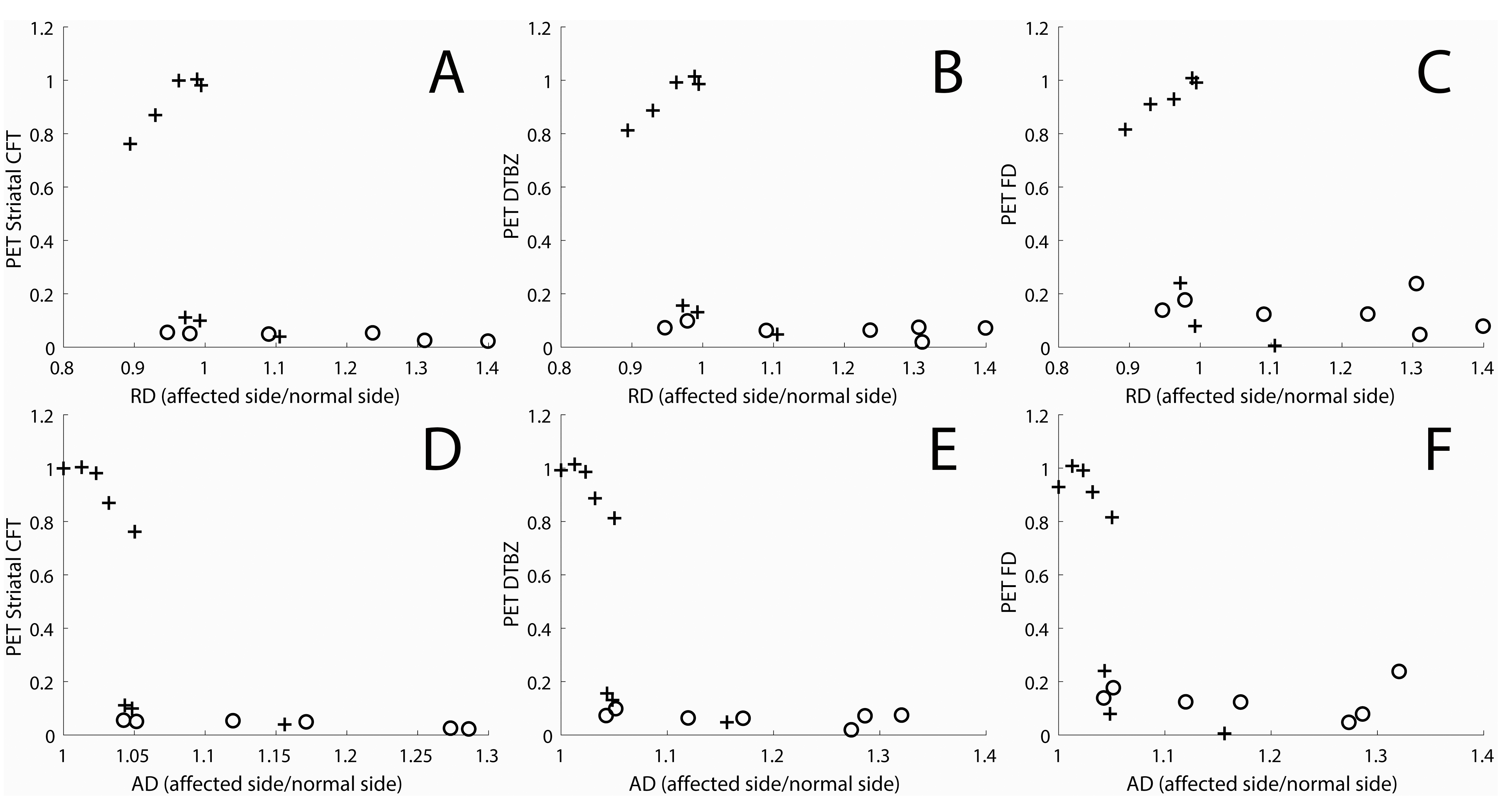

Supplement: S3 Fig — The measures include (A,D) Striatal CFT (a DAT marker), (B,E) DTBZ (a VMAT2 marker), (C,F) FD (a decarboxylase marker). Crosses indicate monkey’s in which there was less than 50% loss of nigral cell bodies, and circles indicate monkeys in which there was a greater than 50% loss of nigral cell bodies. (TIF) [file pone.0202201.s003.tif]
